# Supplementary material for: Environmental pH and peptide signaling control virulence of Streptococcus pyogenes via a quorum-sensing pathway
Source: Nat Commun. 2019 Jun 13;10:2586. doi: 10.1038/s41467-019-10556-8 (PMC6565748; doi:10.1038/s41467-019-10556-8)
Supplement: Supplementary file 1 — Supplementary Information [file 41467_2019_10556_MOESM1_ESM.pdf]

# Supplementary Information

a

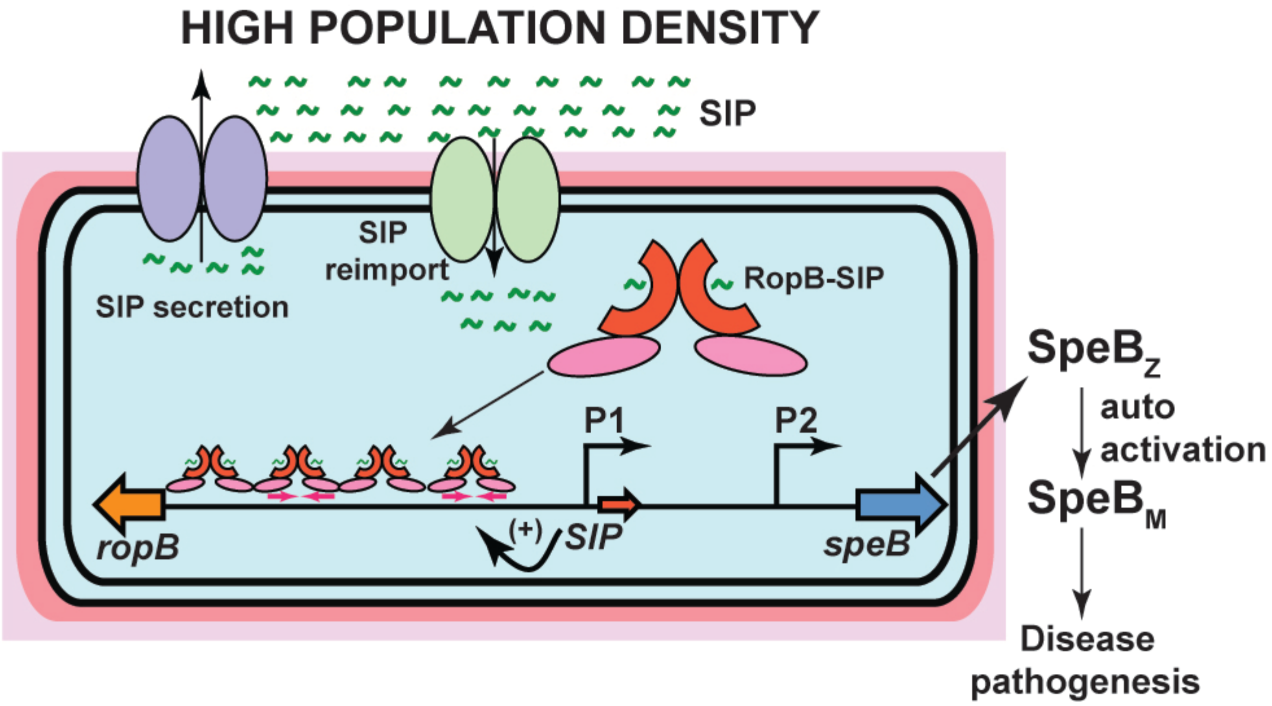


**Supplementary Figure 1. a** Proposed model for mechanism of SIP-dependent intercellular communication and GAS virulence regulation. At high cell density, SIP is produced, secreted, and reimported into the cytosol. The reinternalized SIP interacts with intracellular RopB, and aids high-affinity RopB-DNA interactions and RopB polymerization. The oligomeric RopB bound to *speB* promoter activates *SIP* expression, which results in robust induction of SIP production by a positive feedback mechanism. The SIP-dependent upregulation of *speB* leads to abundant secretion of SpeB in its zymogen form (SpeB$_Z$). Subsequently, SpeB$_Z$ undergoes auto-activation steps to become active, mature SpeB (SpeB$_M$). The SpeB$_M$-mediated proteolytic cleavage of host and GAS proteins facilitates host tissue damage, and disease dissemination. The *ropB* and *speB* genes are divergently transcribed. The block arrows indicate the coding regions of *ropB*, *speB*, and *SIP*. The angled arrows above the line indicate two transcription start sites for *speB*, designated P1 and P2. The pseudo-inverted repeats containing the RopB-binding site within the *speB* promoter are marked by arrows and colored

in red. $SpeB_Z$ indicates the zymogen form of SpeB protease, whereas $SpeB_M$ indicates the mature form of SpeB protease.

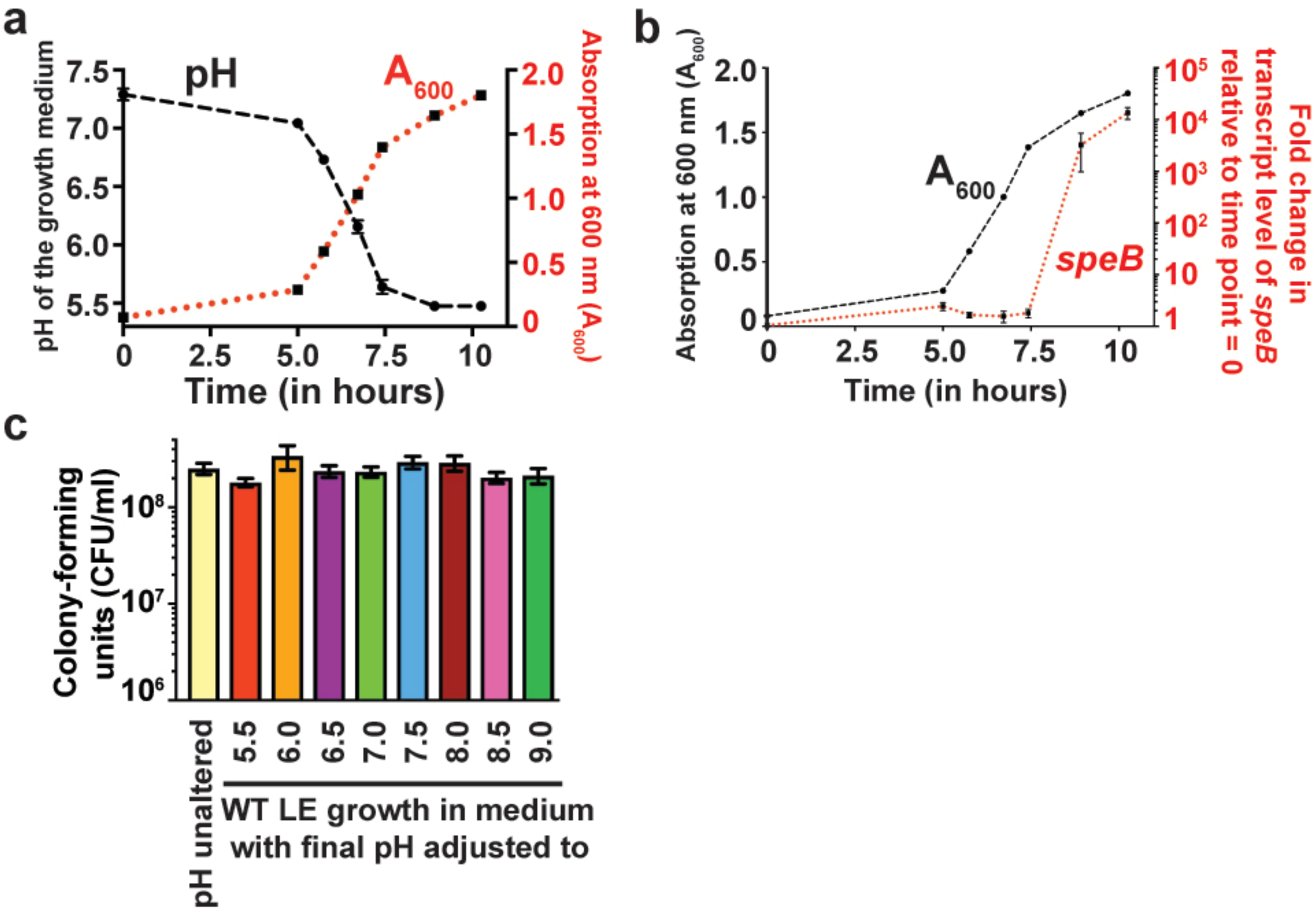


**Supplementary Figure 2. Environment acidification controls *speB* expression. a** The alterations in environmental pH during GAS growth. The correlation between the pH of THY broth during GAS growth and GAS growth kinetics are shown. MGAS10870 strain was grown in THY broth and samples were collected at the indicated time points to determine pH. Left Y axis represents pH of the growth medium at indicated time points. Right Y axis represents the absorption at wavelength 600 nm ($A_{600}$). **b** The correlation between $A_{600}$ during GAS growth and *speB* transcript levels are shown. MGAS10870 strain was grown in THY broth and samples were collected at the indicated time points to determine $A_{600}$, and *speB* transcript levels. Left Y axis represents absorption at wavelength 600 nm ($A_{600}$). Right Y axis represents the fold change in *speB* transcript levels at indicated time points, as measured by qRT-PCR. Fold change in transcript levels relative to starting culture (time point *t*=0 h) is shown. Data graphed are mean ± standard deviation for three biological replicates. **c** The pH of growth medium does

not affect GAS viability. The WT GAS was grown in THY broth to late-exponential growth phase (LE, $A_{600}$ ~ 1.5). Cells were harvested and resuspended in fresh THY medium adjusted for the indicated pH. After 1 h incubation, cells were plated, incubated overnight, and colony-forming units were determined. Data graphed are mean $\pm$ standard deviation for three biological replicates.

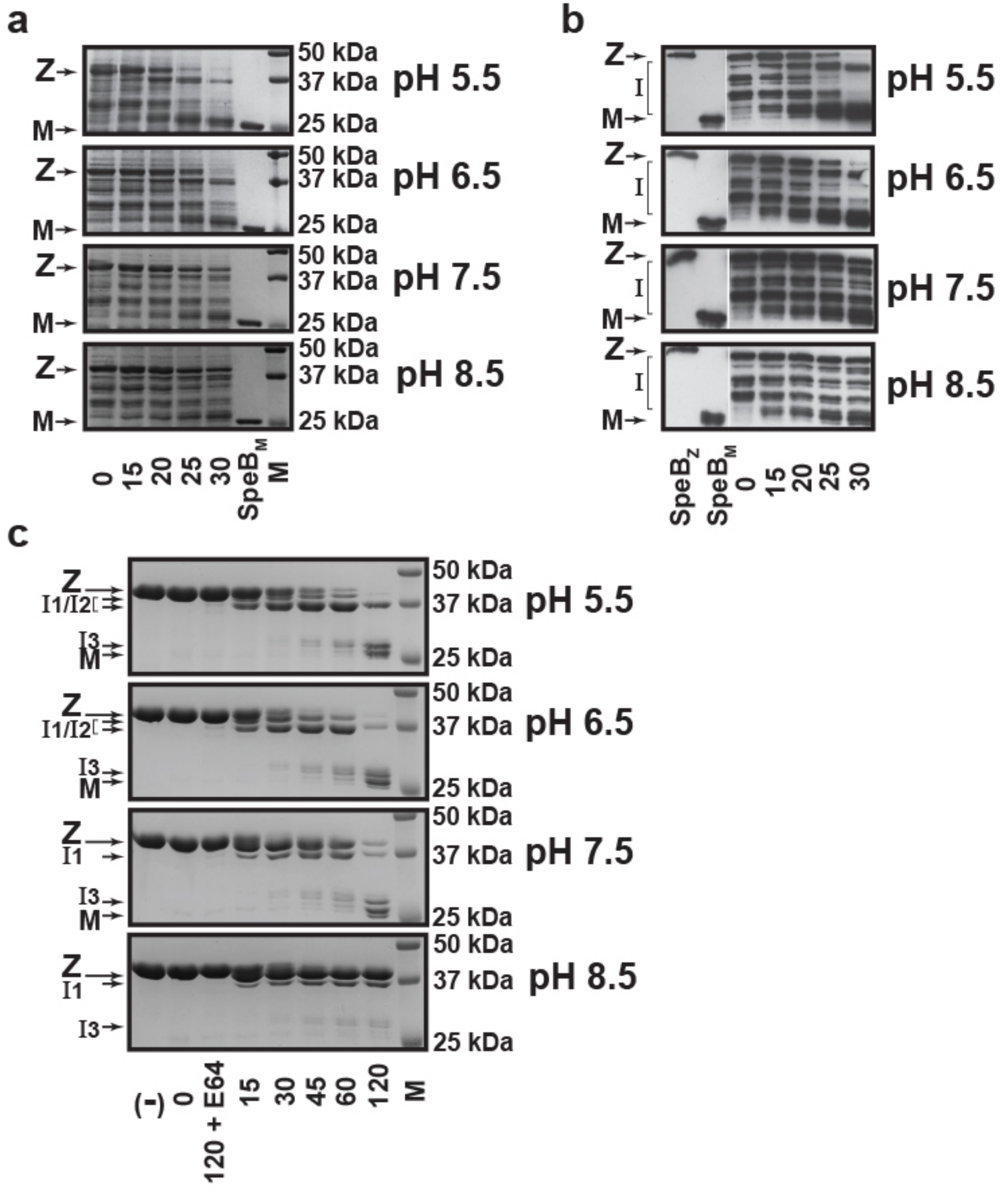


**Supplementary Figure 3. Environmental pH influences auto-activation and proteolytic activities of SpeB.** SDS-PAGE **(a)** and Western immunobloting **(b)** analysis of auto-activation of $SpeB_Z$ to SpeBM. Purified recombinant $SpeB_Z$ (0.25 mg/ml) was incubated in reaction

conditions with different pH and samples collected at indicated time points (in minutes) were analyzed. Samples were probed with anti-SpeB polyclonal rabbit antibody and detected by chemiluminescence **(b)**. The zymogen (Z), intermediate products (I), and mature form of SpeB (M) are indicated by arrows on the left. The masses of molecular weight markers (in kilo daltons, kDa) are shown on the right. **c** SDS-PAGE analysis of proteolytic cleavage of catalytically inactive SpeB mutant (C192S) in its zymogen form by recombinant $SpeB_M$. Purified C192S zymogen at 0.25 mg/ml was incubated with 0.1 µg of $SpeB_M$ in different pH conditions and samples collected at indicated time points (in minutes) were analyzed by SDS-PAGE. The zymogen (Z), intermediate products (I1-3), and mature form of SpeB (M) are indicated by arrows on the left. The masses of molecular weight markers (in kilo daltons, kDa) are shown on the right.

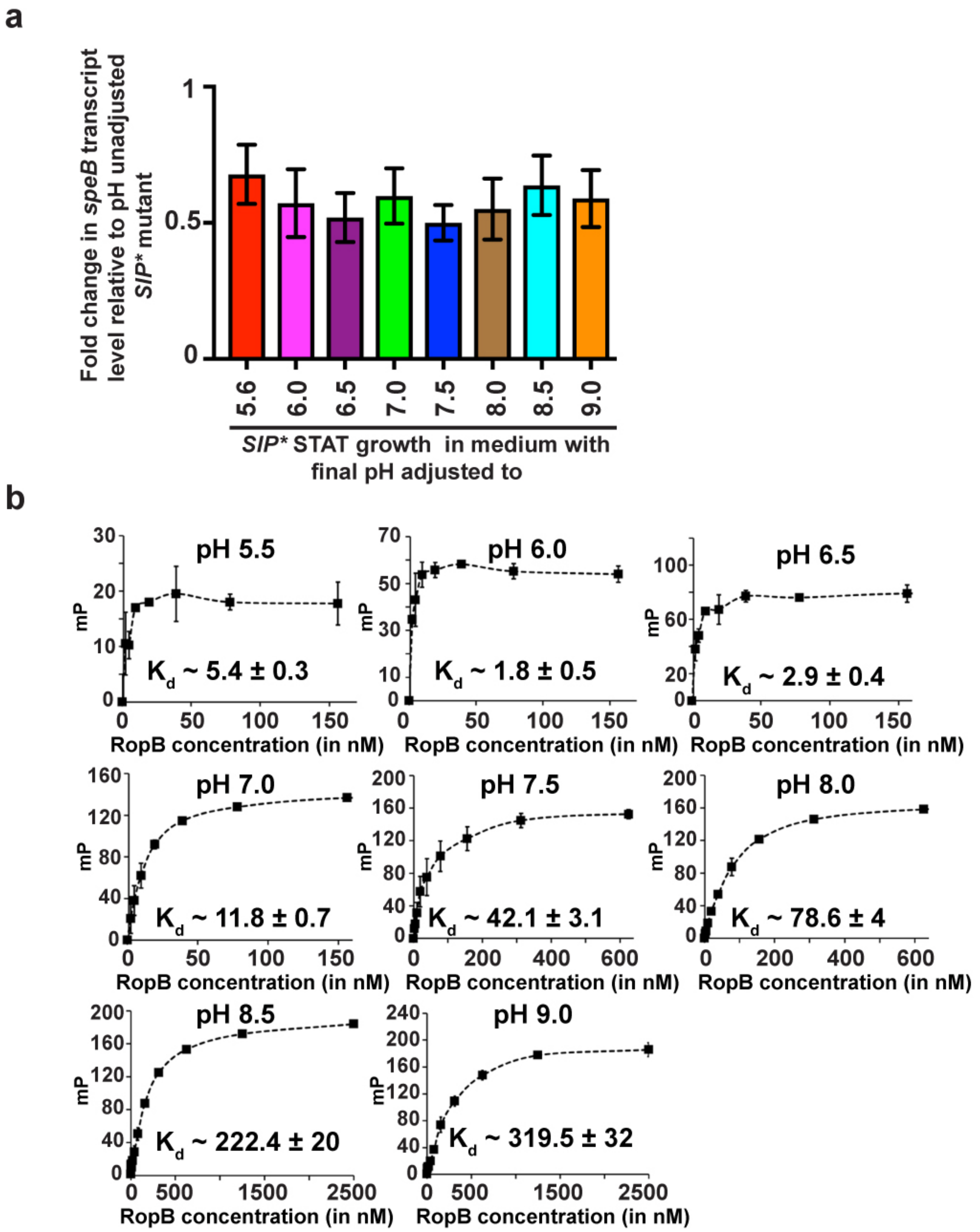
a
Fold change in speB transcript level relative to pH unadjusted SIP* mutant
1
0.5
0
5.6
6.0
6.5
7.0
7.5
8.0
8.5
9.0
SIP* STAT growth in medium with final pH adjusted to
b
pH 5.5
Kd ~ 5.4 ± 0.3
pH 6.0
Kd ~ 1.8 ± 0.5
pH 6.5
Kd ~ 2.9 ± 0.4
pH 7.0
Kd ~ 11.8 ± 0.7
pH 7.5
Kd ~ 42.1 ± 3.1
pH 8.0
Kd ~ 78.6 ± 4
pH 8.5
Kd ~ 222.4 ± 20
pH 9.0
Kd ~ 319.5 ± 32
mP
RopB concentration (in nM)

**Supplementary Figure 4. Environmental acidification influences SIP recognition by RopB. a** The *SIP** mutant strain was grown to early stationary phase (STAT, $A_{600}$ ~ 1.7). Cells were resuspended in THY adjusted to indicated pH and the fold-changes in *speB* transcript levels relative to the unsupplemented *SIP** mutant strain are shown. **b** Analysis of the binding between purified RopB and fluoresceinated SIP in the indicated binding pH conditions by a fluorescence polarization (FP) assay.

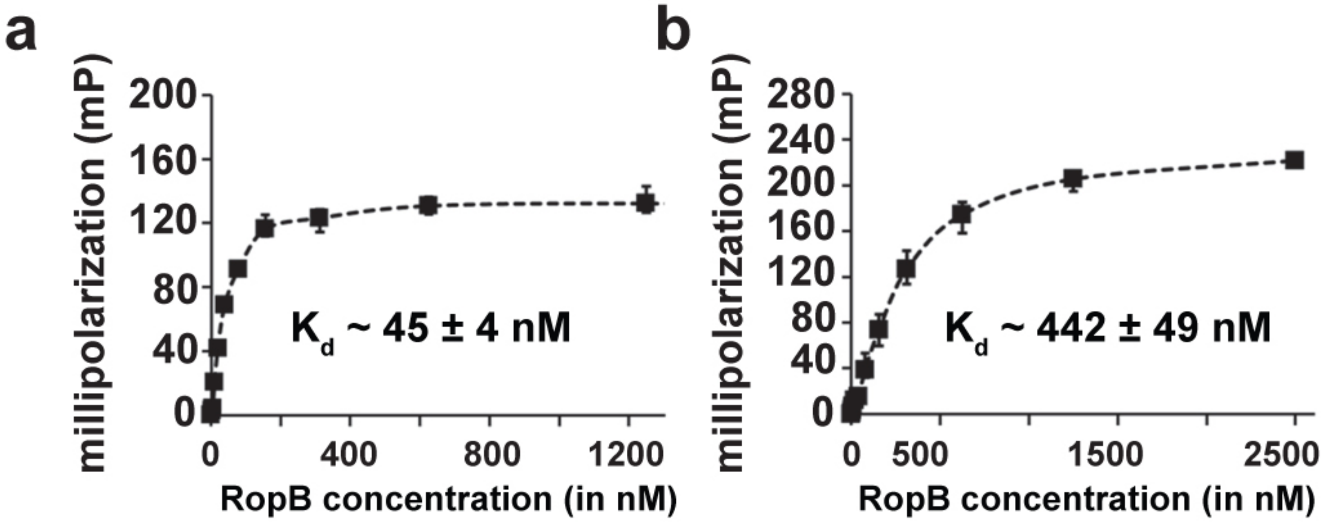


**Supplementary Figure 5.** Analysis of the binding between purified recombinant RopB-CTD and fluoresceinated SIP **(a)** or scrambled non-specific peptide (SCRA) **(b)** by FP assay.

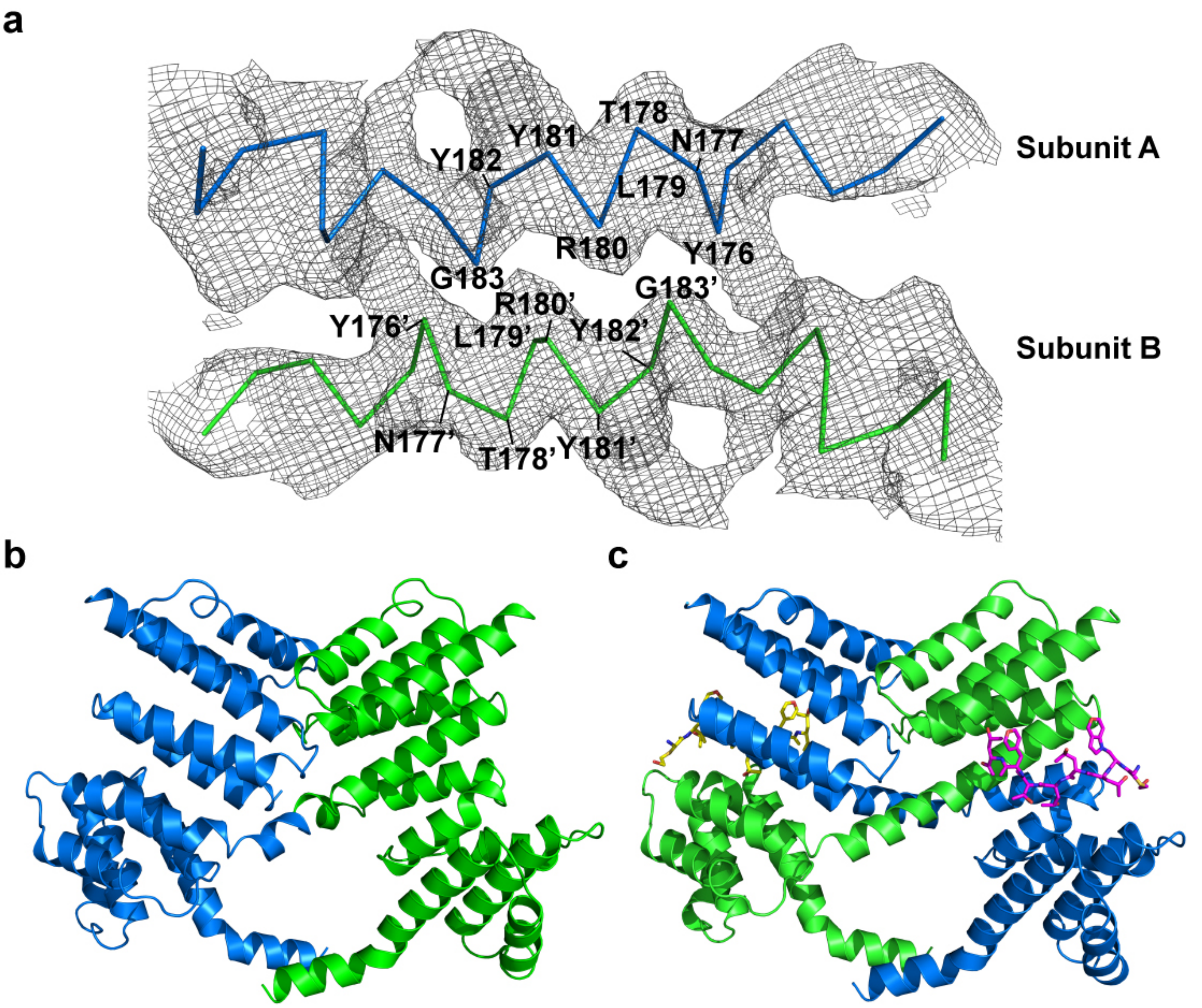


**Supplementary Figure 6. a** The composite omit map of the region corresponding to RopB amino acids 170-191 unambiguously demonstrate the presence of single long helix in the RopB-CTD-SIP structure. The map is shown at the 1-sigma level. The electron density for a RopB-CTD-SIP subunit (subunit A) and its symmetry mate (subunit B) is shown. The corresponding helices in each RopB-CTD subunit are shown as lines and the individual subunits are color coded in blue and green, respectively. The amino acids in the helices are labeled and numbered. The ‘ indicates the amino acid residues from the second subunit (subunit B) of RopB-CTD. Ribbon representation of the structures of apo RopB-CTD with the helix-loop-helix arrangement (**b**) and RopB-CTD-SIP (**c**) with a continuous helix in the region

containing RopB amino acids 159-200 are shown. The individual subunits of a RopB-CTD dimer are color coded in blue and green, respectively. The SIP molecule bound to each RopB-CTD subunit (**c**) is shown as sticks and colored in pink and yellow, respectively.

**a**

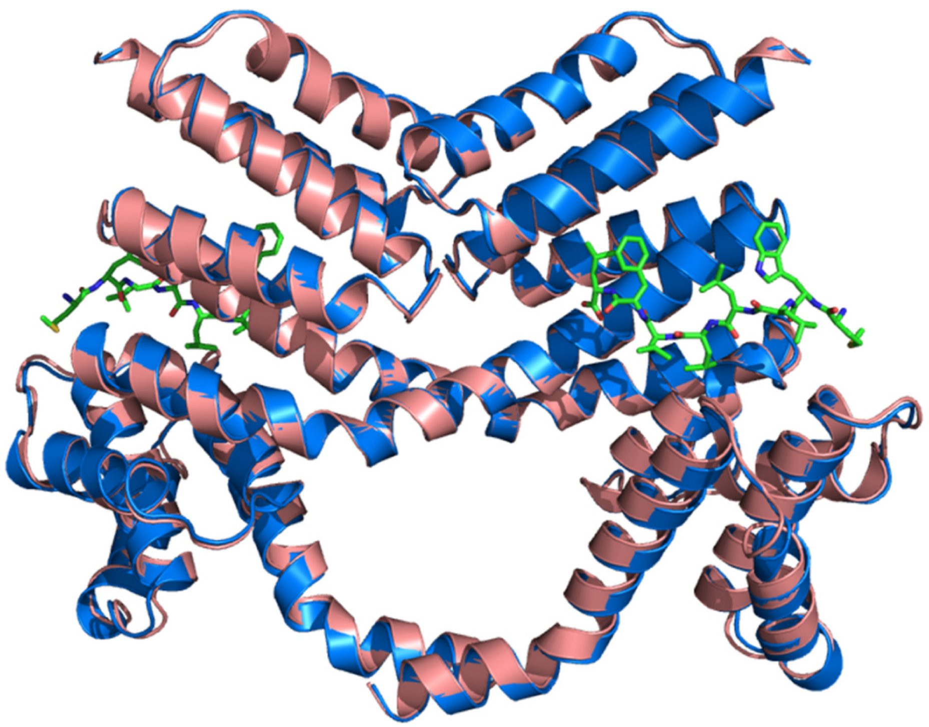

**b**

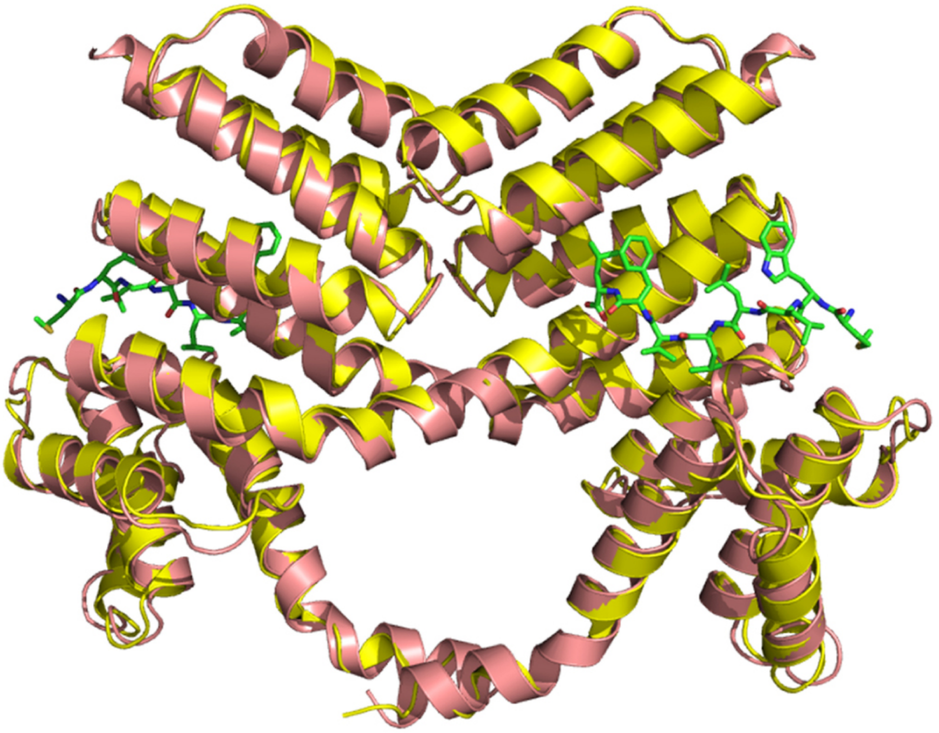

**Supplementary Figure 7. a** The SIP-bound RopB-CTD dimer can be superimposed to SIP-free RopB-CTD dimers with a root mean square deviation (r.m.s.d) of 0.6 Å. **b** The SIP-bound

RopB-CTD dimer and apo RopB-CTD dimer (PDB code:5DL2) can be superposed with a r.m.s.d of 0.7 Å. The SIP-bound RopB-CTD dimer is colored in pink, the SIP-free dimer is colored in blue, and the apo RopB-CTD dimer is colored in yellow.

**a**

| SIP amino acid | RopB amino acid | Distance (in angstroms) |
|---|---|---|
| Trp 2 - CG | Gln 199-CD | 3.9 |
| Trp 2 - CD2 | Gln 199-CD | 3.7 |
| Trp 2 - CE2 | Gln 199-CD | 3.9 |
| Trp 2 - CE3 | Gln 199-CD | 3.9 |
| Leu 5 - CD2 | Phe 155-CD1 | 2.6 |
| Leu 5 - CD2 | Phe 155-CE1 | 3.4 |
| Leu 5 - CD2 | Phe 155-CG | 3.1 |
| Phe 7 - CD1 | Met 267-CE | 3.9 |
| Phe 7 - CE1 | Met 267-CB | 3.4 |
| Phe 7 - CE1 | Met 267-CG | 3.2 |
| Phe 7 - CE1 | Met 267-CE | 3.9 |
| Phe 7 - CZ | Met 267-CB | 3.7 |
| Phe 7 - CZ | Met 267-CG | 4.0 |
| Leu 8 - CD2 | Phe 268-CD1 | 3.3 |
| Leu 8 - CD2 | Phe 268-CE1 | 3.2 |
| Leu 8 - CD2 | Phe 268-CZ | 3.7 |
| Leu 8 - CB | Val 191-CG2 | 4.5 |
| Leu 6 - O | Asn 192-ND2 | 2.4 |
| Leu 8 - OXT | Tyr 224-OH | 4.2 |
| Leu 8 - O | Arg 188-NH1 | 2.2 |

**b**

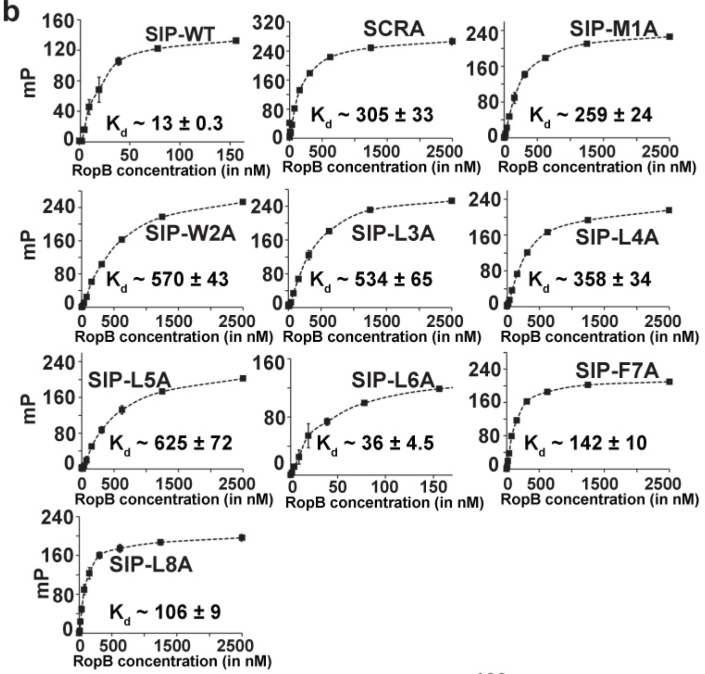


**c**

| Binding buffer pH | $K_d$ (in nM) | [a]Fold change reduction in $K_d$ |
|---|---|---|
| SIP-WT | 57 ± 15 | - |
| Apo-RopB | 412 ± 40 | 7 |
| SCRA | 186 ± 39 | 3 |
| SIP-M1A | 147 ± 33 | 2.6 |
| SIP-W2A | 180 ± 23 | 3.2 |
| SIP-L3A | 221 ± 38 | 4 |
| SIP-L4A | 177 ± 30 | 3 |
| SIP-L5A | 188 ± 17 | 3.3 |
| SIP-L6A | 51 ± 10 | - |
| SIP-F7A | 131 ± 30 | 2.3 |
| SIP-L8A | 191 ± 31 | 3.3 |

[a] Fold change is the ratio of the $K_d$ for DNA with RopB-SIP WT to the $K_d$ with SIP mutant peptides.

**d**

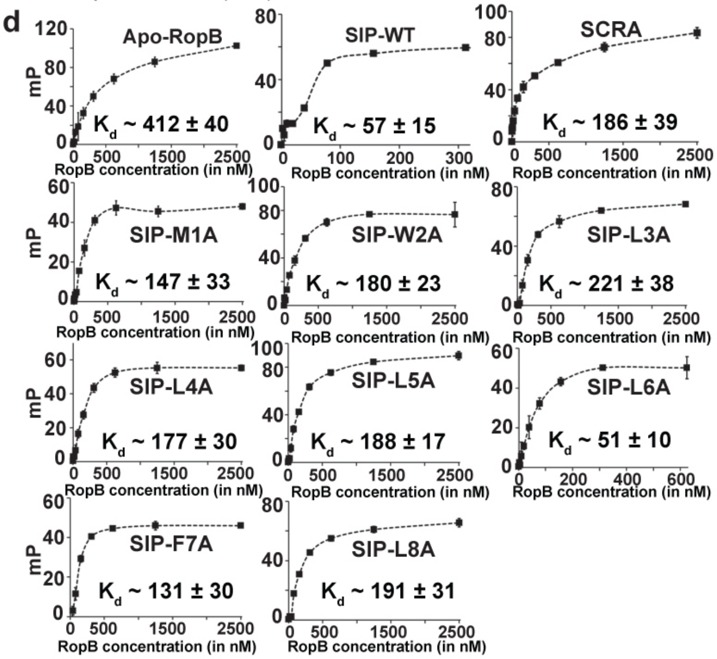


**e**

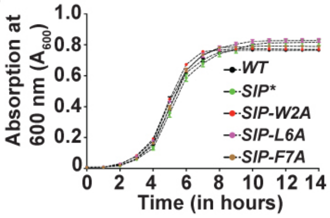


**Supplementary Figure 8. Significance of the individual amino acids of SIP to RopB-peptide and RopB-DNA interactions. a** Table showing the distance between the amino acids of RopB that make contacts with the amino acids of SIP. **b** Analysis of the binding between purified RopB and fluoresceinated SIP-WT or SIP mutants containing single alanine substitutions by fluorescence polarization (FP) assay. **c** RopB-DNA binding constants for apo-RopB or RopB complexed with indicated SIP peptides as assessed by FP assay. **d** Analysis

of the binding between the FITC-labeled oligoduplex containing the putative RopB binding site and apo-RopB or RopB bound to indicated SIP peptides by FP assay. **e** Growth kinetics of the indicated strains in THY broth. Three biological replicates were grown and the graph represents mean ± standard deviation.

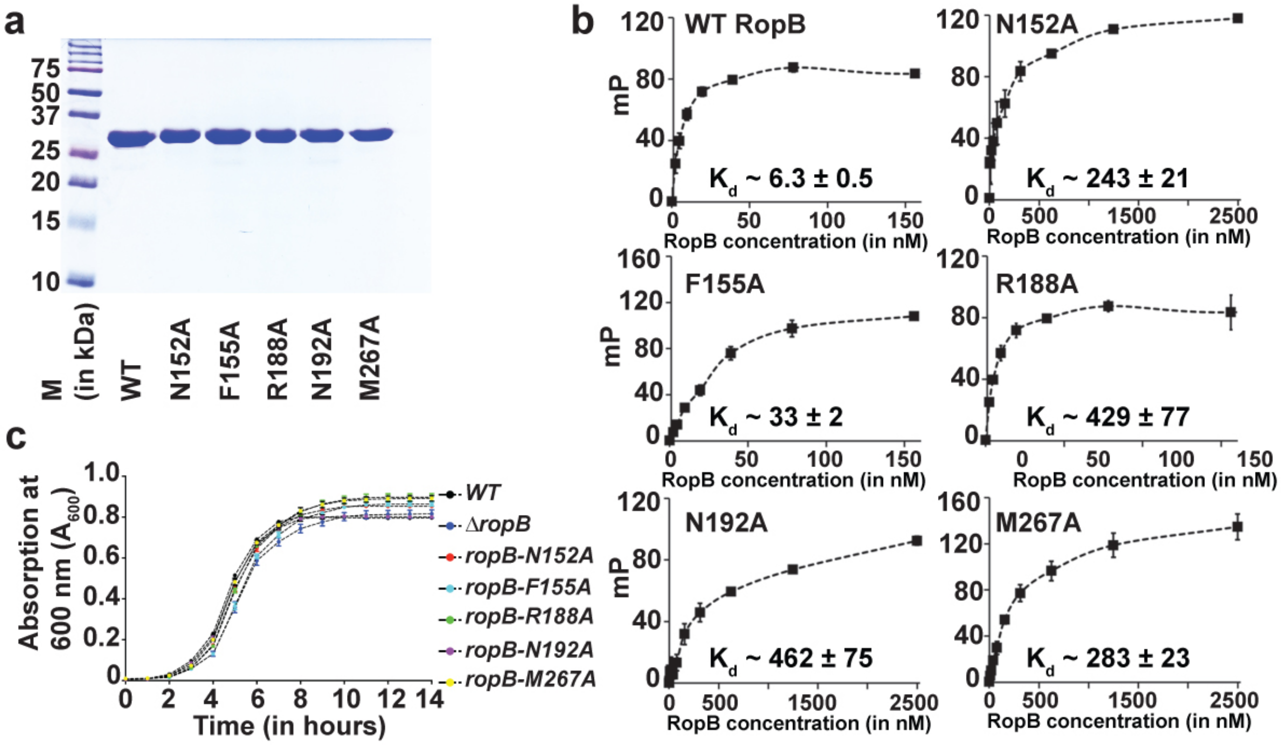


**Supplementary Figure 9. Significance of SIP-contacting residues of RopB to RopB-peptide interactions. a** SDS-PAGE analysis of the purified recombinant WT and mutant RopB proteins. The masses of the molecular weight marker (M, in kilodaltons) are labeled. **b** Analysis of the binding between fluoresceinated SIP and purified WT or mutant RopB by FP assay. **c** Growth kinetics of the indicated strains in THY broth. Three biological replicates were grown and the graph represents mean ± standard deviation.

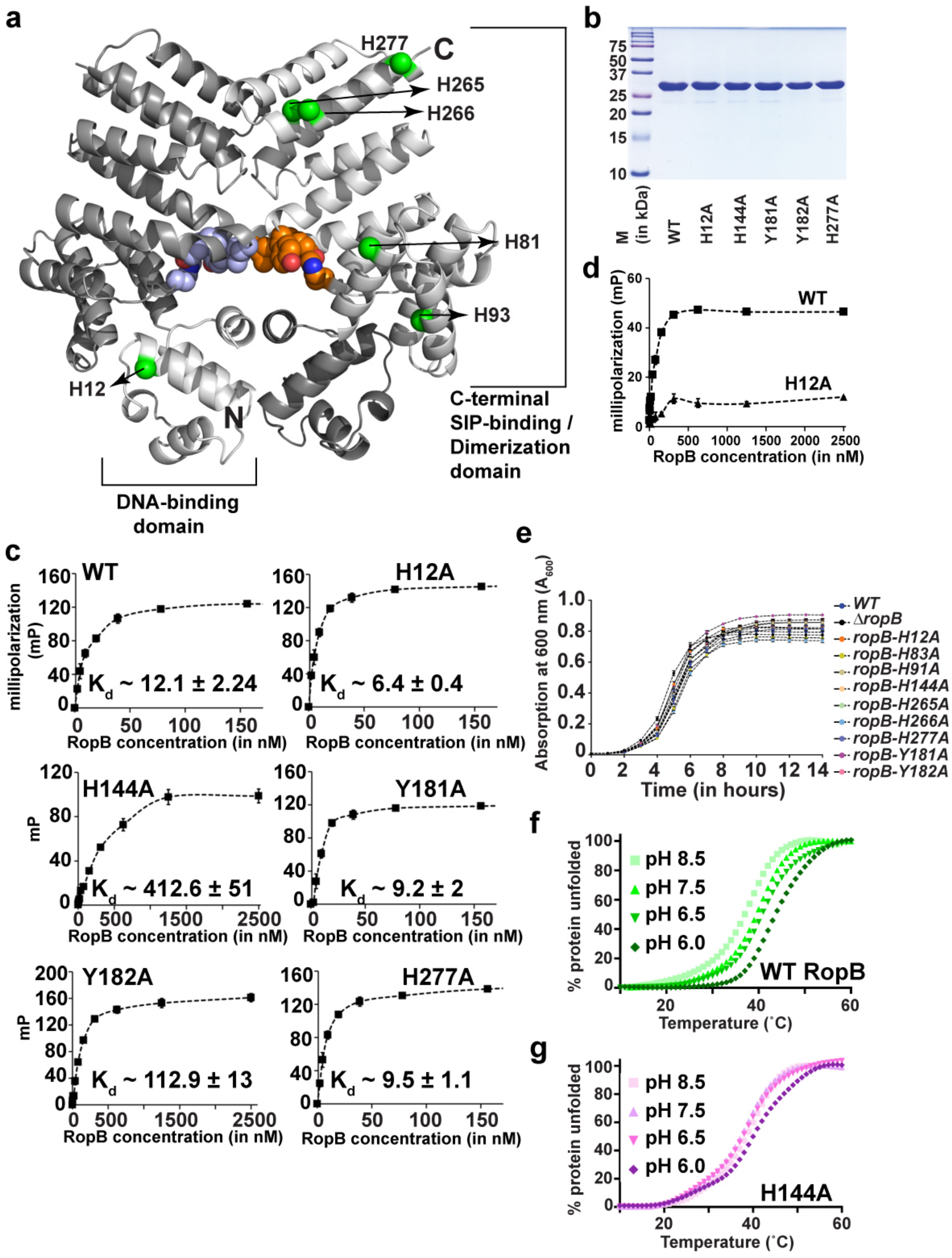
a
H277
C
H265
H266
H81
H93
H12
N
C-terminal SIP-binding / Dimerization domain
DNA-binding domain
b
M (in kDa)
WT
H12A
H144A
Y181A
Y182A
H277A
c
WT
K$_d$ ~ 12.1 ± 2.24
H12A
K$_d$ ~ 6.4 ± 0.4
H144A
K$_d$ ~ 412.6 ± 51
Y181A
K$_d$ ~ 9.2 ± 2
Y182A
K$_d$ ~ 112.9 ± 13
H277A
K$_d$ ~ 9.5 ± 1.1
millipolarization (mP)
mP
RopB concentration (in nM)
d
millipolarization (mP)
WT
H12A
RopB concentration (in nM)
e
Absorption at 600 nm (A$_{600}$)
Time (in hours)
WT
ΔropB
ropB-H12A
ropB-H83A
ropB-H91A
ropB-H144A
ropB-H265A
ropB-H266A
ropB-H277A
ropB-Y181A
ropB-Y182A
f
% protein unfolded
pH 8.5
pH 7.5
pH 6.5
pH 6.0
WT RopB
Temperature (˚C)
g
% protein unfolded
pH 8.5
pH 7.5
pH 6.5
pH 6.0
H144A
Temperature (˚C)

**Supplementary Figure 10. Characterization of RopB histidines for SIP interactions. a** The three-dimensional model of full-length RopB dimer predicted by I-TASSER (https://zhanglab.ccmb.med.umich.edu/I-TASSER/) is shown. Two parameters were used to estimate the accuracy of the predicted model: the template-modeling score (TM-score) and the confidence score (c-score). The predicted full-length RopB model has a high c-score of 0.62, indicating the accuracy of the prediction, and a very reliable TM score of 0.8 ± 0.09, demonstrating the high degree of topological match of the model to the template. The individual subunits of RopB dimer are color-coded in dark and light grey. The N- and C-termini of one subunit is marked as N and C, respectively. The N-terminal DNA-binding and C-terminal SIP-binding/dimerization domains of RopB are marked and labeled. The main chain atoms of surface-exposed histidines in one subunit of RopB are shown as green spheres and labeled. The side chains of H144, Y176, and Y182 at the base of the SIP-binding pocket from each subunit of a RopB dimer are shown as spheres and the side chains from two subunits are color-coded in orange and purple, respectively. **b** SDS-PAGE analyses of purified recombinant WT and mutant RopB proteins. The masses of molecular weight markers (M) in kilodaltons (kDa) are marked. **c** Analysis of the binding between fluoresceinated SIP and purified WT or mutant RopB by FP assay. **d** Analysis of the binding between the FITC-labeled oligoduplex containing the putative RopB binding site and WT-RopB or H144A bound to SIP by FP assay. **e** Growth kinetics of the indicated strains in THY broth. Three biological replicates were grown and the graph represents mean ± standard deviation. Thermal stability of recombinant WT RopB **(f)** and H144A mutant **(g)** proteins as determined by a thermofluor assay.

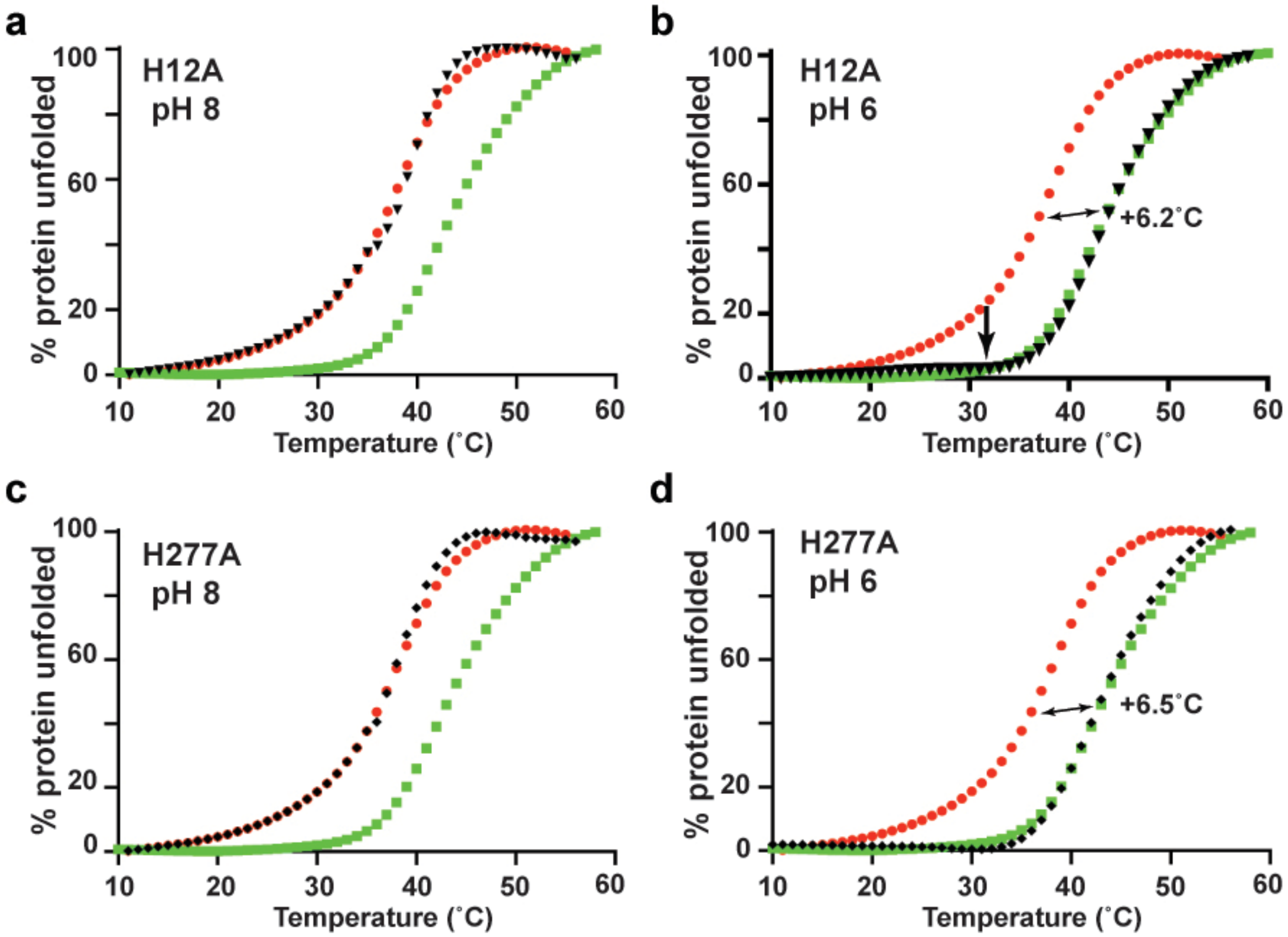


**Supplementary Figure 11. The non-pH sensing histidines in RopB are not involved in pH-dependent stabilization.** Thermal stability of recombinant H12A *(a-b)* and H277A mutant *(c-d)* proteins in high (pH 8) and low pH (pH 6) as determined by a thermofluor assay.

```
                                  H12
S. pyogenes       ---------MEIGETVEFIRHSKNISIKQVCGDYLTRQTYYRFIKNNLDIS
S. porcinus       ---------MELGETVEFIRHSKNIPIKQVCGDYLTRQTYHRFIKNNLDIS
S. psuedoporcinus ---------MELGETVEFIRHSKNIPIKQVCGDYLTRQTYHRFIKNNLDIS
S. salivarius     MLHSMEVAMNFGQAIDHLRRQKHFSVKQICGNYLSRQTYYRFVNNEVDIS
L. pentosus       ---------MELGETLEFIRRNKNISLKELCGTTLSRQNYYRIVHGQVNTS
Enterococcus sp.  ---------MEIGETIEFLRKQKGLTIKDVCGKELSRQTFYKFVKGETSTS
L. aviarius       -----MEEN-YGKIVSNIRIAKGVPIGKLISGICSRTAYRNFVMNRTGTS
E. faecalis       -----METKDWGKTISYIRKNKNIPIKQVIGEKITRSAYSRFASGQTNTS
L. reuteri        ------MKKTLGETIGEIRKNKRIPVSSLCSGVIAKSTYTRCVQNKTSIS
                             *: :   :*  *  ..: .: .    ::  : .    .. . *
                                                             H81
S. pyogenes       SKKLLYILDNLNVNVDEFLFISNNFKQYKEFIDMDTAKHYFECRNIEGLN
S. porcinus       STKLIYILDNLNVNVDEFLFISNNFKRYQEFIDMDTAKHYFENQDTTGLH
S. psuedoporcinus STKLLYILDNLNVNVDEFLFISNNFKRYQEFTDMDTAKQYFETQNISGLN
S. salivarius     STKLFYLLNHLNVNVDEFLFICNNFQLEKEFTDMEKIKIYFEKNNIVGLQ
L. pentosus       INTFKFILDQLHVNFDEFYFIKNNFRQDKIFADMNKVKTFFDKGDLKSLD
Enterococcus sp.  ITNFSYILDKLCVEFDEFLLIQNGYEEKEYIKIMKSLKKLFESKNASKIS
L. aviarius       VDNFMKLLAKLHVSYTEFKYIANGFETNYEQRYVIDLQKAIAQGDLKRLD
E. faecalis       IDNFFFMQNLHINFEEFIYIQNKYELDKYQKLLKKAQVATHQKNIKELE
L. reuteri        TEIFFEFLTNLNVTFEEFEYIRNDYSKDIYIELMEQIKVSFESRNLIKLK
                     :  :: :* :    **   * * :         :   :       :   :
H93
S. pyogenes       HILDSYKDS---KSTKEK--NLFALVKVLLATLTE-EDCLTERTYLSNYL
S. porcinus       QILTSYKDS---KSTKEK--NLFALVKVLLAMLTN-EDCLKERLYLSNYL
S. psuedoporcinus HILESYKDS---KSTEEK--NLFALVKVLLAMLTN-EDCLKERLYLSNYL
S. salivarius     NLLTSYSSQ---INVKEK--IIYALISSLLGRLTN-TSSCTEEKILRDYL
L. pentosus       KMIAKYLEL---KQINQSYLHMYCLINVLKHKLSN-HASGPCEALLRDYL
Enterococcus sp.  KIKQYCFDN---KDMNQKFLHLYYISDILLAKILD-IPFEKSATLMQKYL
L. aviarius       DLLQRTLNYCAIYENDEKYRHLACITQLTIDKVKRVPLDEDARQVVIDYL
E. faecalis       KIKNRFDNYAKVTEYIEP-LHLKCIITLTINKLKKEPYDKNAKQIITEYL
L. reuteri        EIKKICNQKD--KKHIIKFHHLNLLCDLLIQKMIDPSSELTQSNELITYL
                  .:      .     .       :  :       :              :  **
      H144                            Y176   Y182  E185
S. pyogenes       INIETWSHYETVLFNNCMFILESCFIEMVFSKVIVNLDKYNTLRYYGNES
S. porcinus       VNIETWSHYETVLFNNCMFIFDSNFIEIIFSKVILNLDKYNTLRCYGNES
S. psuedoporcinus VNMETWSHYETVLFNNCMFIFDSNFIEIIFSKVILNLDRYNTLRCYGNES
S. salivarius     INIETWTHYETVLFNNAMFIFDSEFIELIFRKINLNLEKYSTLRYYGNES
L. pentosus       TNVETWTHYETVLFNNCMFIFSTEFIDVTLSKSLHNLSMYSTLRKYGNES
Enterococcus sp.  IGVNSWTHYELVLFNNIMFVFNSEMIDAIINKSTSNLEKYVQLKGYGNEN
L. aviarius       IECETWTHYELMMFNNAMFAFSFDQIRMFREKVIHNLEKYQNLRIYGSES
E. faecalis       EQCESWMHYELVLFNNAMFIFDLNLIKVMKRKVIHNLEKYQNLRSYGSES
L. reuteri        LRVNQWYHYELILFNNCLFVCSPDLIDIILKNIVKNYTQYLDLSKYGNEL
                     : * *** ::*** :*  .   *     :   *   *  *   **.*

S. pyogenes       IRMFVNMLILFIQRQEYDKASEILAKIEDYQLNDDCLYERCCVSFFDGII
S. porcinus       IRMFVNMLILFIQRQEYQKAAEILAQIKNYSLNEDCIYERCCIAFFEGII
S. psuedoporcinus IRMFVNMLILFIQREEYHKAADILAQIKNYNLNEDCLYERCCISFFEGII
S. salivarius     IRMFINMIILYIDRQDIRRAKEALQIAKNFKINDDCMYEKSCIVFFNEVL
L. pentosus       FRMLTNVLILFIEREEFERATFILNKLHQNRLTDDLLFEKACLEFFENAM
Enterococcus sp.  IRMLLNVCFLLLQRKELKKLRKYFNIIKEIDLSEDAIFERCSILFLEALI
L. aviarius       FRVLINMLMVFIENQSYQDIRIMMGLINNYQLNEDMLFEETLRLYFTGIL
E. faecalis       FRILINILMLFLEHSEFNEARLLIDEIDEFHLHGDMFFEKVLRMYFTGLV
L. reuteri        TRVLVNIYTFYLNHFQFEKLIKINTLIQNIPLNEDCLFERTAVLFFDGVH
                   *:: *:  . ::. .            .:  :  * ::*.    ::

                                       H265&H266   H277
S. pyogenes       GLIN-GKEGAEQKCVQILEIFQLLNCKTIHHMFQTYLEAIKHKLS-----
```

```
S. porcinus       DIIH-GKEGAERKCEKILQVFELLNCKTIHKMFKAYLKDIKNKVKYA---
S. psuedoporcinus GMIY-GQKGAEQKCEKILQVFQLLNCKTVHKMFKSYLQDIKNKT------
S. salivarius     NLLN-GEISSLKICKSIIKYFNLIGSLSIAAMFESYLDTLIKNNPHITFN
L. pentosus       LLVT-GETTSDAECQRIINLFRELGSDGLAVIFERYLQKVRQ--------
Enterococcus sp.  NFIN-EENNAEKQIINILNVFNTSNSSNMYSMYHDCYIHVKENKNDFQML
L. aviarius       DLINQKIPAGLTKVKQALEVLSILNAKDYYQNLKQYLEQIILKYDLDIKV
E. faecalis       DFISSKGQE-QALLNESLDILKSISKNEYYAVLSTYLTKMKKQYEILD—
L. reuteri        ELLFKGSEDSHKKIKEAILIFDILHCYNFKKMFITLYEQIKTLDLSK---
                   ::              :  :                  :

S. pyogenes       --
S. porcinus       —
S. psuedoporcinus —
S. salivarius     —
L. pentosus       —
Enterococcus sp.  IL
L. aviarius       --
E. faecalis       --
L. reuteri        --
```

**Supplementary Figure 12. The pH sensing histidine and the amino acids participating in the intramoleular interactions at the base of SIP-binding pocket are conserved among RopB-like proteins.** Amino acid sequence alignment of RopB paralogs from gram-positive bacteria. The positions of His-144, Tyr-176, Tyr-182, and Glu-185 are highlighted (in yellow) and labeled above. Amino acids corresponding to non-conserved histidines are highlighted (in grey) and labeled above. Sequences were aligned using the ClustalW alignment program [1].

Supplementary Table 1. Bacterial strains and plasmids used in this study.

| Strain or Plasmid | Description | Reference |
|---|---|---|
| **Strains** | | |
| WT | Invasive isolate MGAS10870, serotype M3 | [2] |
| Δ*ropB* | MGAS10870Δr*opB::aad9* | [3] |
| Δ*speB* | MGAS10870Δ*speB::aad9* | [4] |
| *sip** | Isoallelic mutant strain that has the start codon of *sip* changed to stop codon in parental serotype MGAS10870 | [5] |
| *sip-W2A* | Isoallelic mutant strain that has the alanine substitution at tryptophan at codon 2 of *sip* in parental serotype MGAS10870 | This study |
| *sip-L6A* | Isoallelic mutant strain that has the alanine substitution at leucine at codon 6 of *sip* in parental serotype MGAS10870 | This study |
| *sip-F7A* | Isoallelic mutant strain that has the alanine substitution at phenylalanine at codon 7 of *sip* in parental serotype MGAS10870 | This study |
| *ropB-H12A* | Isoallelic mutant strain that has the alanine substitution at histidine at codon 12 of *ropB* in parental serotype MGAS10870 | This study |
| *ropB-H81A* | Isoallelic mutant strain that has the alanine substitution at histidine at codon 81 of *ropB* in parental serotype MGAS10870 | This study |
| *ropB-H93A* | Isoallelic mutant strain that has the alanine substitution at histidine at codon 93 of *ropB* in parental serotype MGAS10870 | This study |
| *ropB-H144A* | Isoallelic mutant strain that has the alanine substitution at histidine at codon 144 of *ropB* in parental serotype MGAS10870 | This study |
| *ropB-N152A* | Isoallelic mutant strain that has the alanine substitution at asparagine at codon 152 of *ropB* in parental serotype MGAS10870 | This study |

| | | |
|---|---|---|
| *ropB-F155A* | Isoallelic mutant strain that has the alanine substitution at phenylalanine at codon 155 of *ropB* in parental serotype MGAS10870 | This study |
| *ropB-Y181A* | Isoallelic mutant strain that has the alanine substitution at tyrosine at codon 181 of *ropB* in parental serotype MGAS10870 | This study |
| *ropB-Y182A* | Isoallelic mutant strain that has the alanine substitution at tyrosine at codon 182 of *ropB* in parental serotype MGAS10870 | [6] |
| *ropB-R188A* | Isoallelic mutant strain that has the alanine substitution at arginine at codon 188 of *ropB* in parental serotype MGAS10870 | This study |
| *ropB-N192A* | Isoallelic mutant strain that has the alanine substitution at asparagine at codon 192 of *ropB* in parental serotype MGAS10870 | [6] |
| *ropB-H265A* | Isoallelic mutant strain that has the alanine substitution at histidine at codon 265 of *ropB* in parental serotype MGAS10870 | This study |
| *ropB-H266A* | Isoallelic mutant strain that has the alanine substitution at histidine at codon 266 of *ropB* in parental serotype MGAS10870 | This study |
| *ropB-M267A* | Isoallelic mutant strain that has the alanine substitution at methionine at codon 267 of *ropB* in parental serotype MGAS10870 | This study |
| *ropB-H277A* | Isoallelic mutant strain that has the alanine substitution at histidine at codon 277 of *ropB* in parental serotype MGAS10870 | This study |
| *E. coli* DH5α | Host strain for cloning purposes | |
| *E. coli* BL21 (DE3) | Host strain for protein overexpression, *F-, ompT, hsdSB(rB-mB-), gal(λ c I 857, ind1, Sam7, nin5, lacUV-T7 gene1), dcm(DE3)* | |
| **Plasmids** | | |

| | | |
|---|---|---|
| *pJL* | Low-copy number plasmid capable of replication in GAS and *Escherichia* coli, Chloramphenicol resistant<br>Used to generate isoallelic GAS mutants | [7] |
| *pET21b* | Overexpression vector for C-terminally hexahistidine tagged recombinant proteins, $Amp^R$ | Novagen |
| *pET28a* | Overexpression vector for N-terminally hexahistidine tagged recombinant proteins, $Km^R$ | Novagen |

Supplementary Table 2. Primers and probes used in this study

| Primer | Sequence 5' – 3' | Purpose |
|---|---|---|
| *sip-W2A* top | AAAAGGAGGCGCCTACTATGGCGTTATTG TTACTATTTTTGTAG | 5' primer to introduce alanine substitution at tryptophan at position 2 of *sip* |
| *sip-W2A* Bottom | CTACAAAAATAGTAACAATAACGCCATAGT AGGCGCCTCCTTTT | 3' primer to introduce alanine substitution at tryptophan at position 2 of *sip* |
| *sip-L6A* top | CCTACTATGTGGTTATTGTTAGCGTTTTTG TAGTTCCTTTTGCAAA | 5' primer to introduce alanine substitution at leucine at position 6 of *sip* |
| *sip-L6A* Bottom | TTTGCAAAAGGAACTACAAAAACGCTAAC AATAACCACATAGTAGG | 3' primer to introduce alanine substitution at leucine at position 6 of *sip* |
| *sip-F7A* top | CTATGTGGTTATTGTTACTAGCGTTGTAGT TCCTTTTGCAAAATG | 5' primer to introduce alanine substitution at phenylalanine at position 7 of *sip* |
| *sip-F7A* Bottom | CATTTTGCAAAAGGAACTACAACGCTAGT AACAATAACCACATAG | 3' primer to introduce alanine substitution at phenylalanine at position 7 of *sip* |
| *ropB-H12A* top | ACCGTTGAATTCATTAGGGCGTCAAAAA CATTTCGATT | 5' primer to introduce alanine substitution at histidine 12 of *ropB* |

| | | |
|---|---|---|
| *ropB-H12A* Bottom | AATCGAAATGTTTTTTGACGCCCTAATGAA TTCAACGGT | 3' primer to introduce alanine substitution at histidine 12 of *ropB* |
| *ropB-H81A* top | GATATGGATACGGCAAAAGCGTATTTTGA ATGCCGAAAC | 5' primer to introduce alanine substitution at histidine 81 of *ropB* |
| *ropB-H81A* Bottom | GTTTCGGCATTCAAAATACGCTTTTGCCGT ATCCATATC | 3' primer to introduce alanine substitution at histidine 81 of *ropB* |
| *ropB-H93A* top | AACATAGAAGGTTTAAATGCGATCCTTGAT TCTTATAAA | 5' primer to introduce alanine substitution at histidine 93 of *ropB* |
| *ropB-H93A* Bottom | TTTATAAGAATCAAGGATCGCATTTAAACC TTCTATGTT | 3' primer to introduce alanine substitution at histidine 93 of *ropB* |
| *ropB-H144A* top | AATATTGAAACTTGGAGTGCGTATGAGAC TGTGCTTTTT | 5' primer to introduce alanine substitution at histidine 144 of *ropB* |
| *ropB-H144A* Bottom | AAAAAGCACAGTCTCATACGCACTCCAAG TTTCAATATT | 3' primer to introduce alanine substitution at histidine 144 of *ropB* |
| *ropB-N152A* top | GAGACTGTGCTTTTTAATGCGTGTATGTTC ATTTTTGAG | 5' primer to introduce alanine substitution at asparagine 152 of *ropB* |
| *ropB-N152A* Bottom | CTCAAAAATGAACATACACGCATTAAAAAG CACAGTCTC | 3' primer to introduce alanine substitution at asparagine 152 of *ropB* |
| *ropB-F155A* top | TTTAATAATTGTATGGCGATTTTTGAGTCT TGC | 5' primer to introduce alanine substitution at phenylalanine 155 of *ropB* |
| *ropB-F155A* Bottom | GCAAGACTCAAAAATCGCCATACAATTATT AAA | 3' primer to introduce alanine substitution at phenylalanine 155 of *ropB* |
| *ropB-Y181A* top | TACAATACCCTAAGGGCTTATGGGAATGA ATCG | 5' primer to introduce alanine substitution at tyrosine 181 of *ropB* |
| *ropB-Y181A* Bottom | CGATTCATTCCCATAAGCCCTTAGGGTATT GTA | 3' primer to introduce alanine substitution at tyrosine 181 of *ropB* |
| *ropB-Y182A* top | AATACCCTAAGGTATGCTGGGAATGAATC GATTC | 5' primer to introduce alanine substitution at tyrosine 182 of *ropB* |

| | | |
|---|---|---|
| *ropB-Y182A* Bottom | GAATCGATTCATTCCCAGCATACCTTAGG GTATT | 3’ primer to introduce alanine substitution at tyrosine 182 of *ropB* |
| *ropB-R188A* top | GGGAATGAATCGATTGCGATGTTTGTCAA TATGTTG | 5’ primer to introduce alanine substitution at arginine 188 of *ropB* |
| *ropB-R188A* Bottom | CAACATATTGACAAACATCGCAATCGATTC ATTCCC | 3’ primer to introduce alanine substitution at arginine 188 of *ropB* |
| *ropB-N192A* top | ATTCGGATGTTTGTCGCTATGTTGATTTT | 5’ primer to introduce alanine substitution at asparagine 192 of *ropB* |
| *ropB-N192A* Bottom | CAAAATCAACATAGCGACAAACATCCGAA T | 3’ primer to introduce alanine substitution at asparagine 192 of *ropB* |
| *ropB-H265A* top | CTTAATTGCAAAACGATCGCGCATATGTTT CAAACCTAC | 5’ primer to introduce alanine substitution at histidine 265 of *ropB* |
| *ropB-H265A* Bottom | GTAGGTTTGAAACATATGCGCGATCGTTT TGCAATTAAG | 3’ primer to introduce alanine substitution at histidine 265 of *ropB* |
| *ropB-H266A* top | AATTGCAAAACGATCCATGCGATGTTTCAA ACCTACCTA | 5’ primer to introduce alanine substitution at histidine 266 of *ropB* |
| *ropB-H266A* Bottom | TAGGTAGGTTTGAAACATCGCATGGATCG TTTTGCAATT | 3’ primer to introduce alanine substitution at histidine 266 of *ropB* |
| *ropB-M267A* top | CAAAACGATCCATCATGCGTTTCAAACCTA CCTAG | 5’ primer to introduce alanine substitution at methionine 267 of *ropB* |
| *ropB-M267A* Bottom | CTAGGTAGGTTTGAAACGCATGATGGATC GTTTTG | 3’ primer to introduce alanine substitution at methionine 267 of *ropB* |
| *ropB-H277A* top | CTACCTAGAAGCCATTAAAGCGAAACTGT CCTGAGGCTCTTTA | 5’ primer to introduce alanine substitution at histidine 277 of *ropB* |
| *ropB-H277A* Bottom | TAAAGAGCCTCAGGACAGTTTCGCTTTAA TGGCTTCTAGGTAG | 3’ primer to introduce alanine substitution at histidine 277 of *ropB* |
| pET28a-*ropB-H277A* top | CCTAGAAGCCATTAAAGCGAAACTGTCCT GAGATCCGAA | 5’ primer to introduce alanine substitution at |

| | | |
|---|---|---|
| | | histidine 277 of *ropB* in pET28a vector |
| pET28a-*ropB-H277A* Bottom | TTCGGATCTCAGGACAGTTTCGCTTTAAT GGCTTCTAGG | 3’ primer to introduce alanine substitution at histidine 277 of *ropB* pET28a vector |
| *speB*-C192S top | CAACATGCAGCTACAGGAAGTGTTGCTAC TGCAACTGC | 5’ primer to introduce serine substitution at cystein 192 of *speB* |
| *speB*-C192S bottom | GCAGTTGCAGTAGCAACACTTCCTGTAGC TGCATGTTG | 3’ primer to introduce serine substitution at cystein 192 of *speB* |
| *tufA* qRTFwd | CAACTCGTCACTATGCGCACAT | 5’ primer for *tufA* qRT-PCR |
| *tufA* qRTRev | GAGCGGCACCAGTGATCAT | 3’ primer for *tufA* qRT-PCR |
| *tufA* probe | CTCCAGGACACGCGGACTACGTTAAAAA | Probe for *tufA* qRT-PCR |
| *speB* qRTFwd | ACTCTACCAGCGGATCATTTG | 5’ primer for *speB* qRT-PCR |
| *speB* qRTRev | CAGCGGTACCAGCATAAGTAG | 3’ primer for *speB* qRT-PCR |
| *speB* probe | TGCTTCCTTCATGGAAAGTTATGTCGAACA | Probe for *speB* qRT-PCR |

Supplementary Table 3. Data collection and refinement statistics

| | RopB-CTD-SIP |
|---|---|
| **Data collection** | |
| Space group | $P3_212$ |
| Cell dimensions | |
| *a*, *b*, *c* (Å) | 94.21, 94.21, 179.96 |
| α, β, γ (°) | 90, 90, 120 |
| Resolution (Å) | 30.00-3.30 (3.41-3.30) * |
| $R_{sym}$ or $R_{merge}$ [a] | 0.065 (1.772) |
| $I / \sigma I$ | 24.5 (2.4) |
| Completeness (%) | 99.5 (99.1) |
| Redundancy | 20.3 (20.4) |
| Wilson B-factor (Å$^2$) | 93.7 |
| **Refinement** | |
| Resolution (Å) | 29.17 – 3.30 (3.38-3.30) |
| No. reflections | 13279 (975) |
| $R_{work}$ / $R_{free}$ [b] | 0.23-0.26(0.21-0.27) |
| No. atoms | |
| Protein | 3781 |
| SIP | 74 |
| Water | 13 |
| *B*-factors | |
| Protein | 175.7 |
| SIP | 236.9 |
| Water | 151.1 |
| R.m.s. deviations | |
| Bond lengths (Å) | 0.009 |
| Bond angles (°) | 1.01 |

Single crystal was used for the data collection.
*Statistics for the highest-resolution shell are shown in parentheses.

[a]$R_{sym} = \sum\sum|I_{hkl}-I_{hkl(j)}|/ \sum I_{hkl}$, where $I_{hkl(j)}$ is the observed intensity and $I_{hkl}$ is the final average intensity value.
[b]$R_{work} = S||F_{obs}| - |F_{calc}||/S|F_{obs}|$ and $R_{free} = S||F_{obs}| - |F_{calc}||/S|F_{obs}|$; where all reflections belong to a test set of 5% randomly selected reflections. Values in parentheses are for the highest resolution shell.

## Supplementary References


1 Larkin, M. A. *et al.* Clustal W and Clustal X version 2.0. *Bioinformatics* **23**, 2947-2948, 2007.

2 Beres, S. B. *et al.* Molecular complexity of successive bacterial epidemics deconvoluted by comparative pathogenomics. *Proc. Natl. Acad. Sci. USA* **107**, 4371-4376, 2010.

3 Carroll, R. K. *et al.* Naturally occurring single amino acid replacements in a regulatory protein alter streptococcal gene expression and virulence in mice. *J. Clin. Invest.* **121**, 1956-1968, 2011.

4 Shelburne III, S. A. *et al.* An amino-terminal signal peptide of Vfr protein negatively influences RopB-dependent SpeB expression and attenuates virulence in *Streptococcus pyogenes*. *Mol. Microbiol.* **82**, 1481-1495, 2011.

5 Do, H. *et al.* Leaderless secreted peptide signaling molecule alters global gene expression and increases virulence of a human bacterial pathogen. *Proc. Natl. Acad. Sci. USA* **114**, E8498-E8507, 2017.

6 Makthal, N. *et al.* Structural and functional analysis of RopB: a major virulence regulator in *Streptococcus pyogenes*. *Mol. Microbiol.* **99**, 1119-1133, 2016.

7 Li, J., Kasper, D. L., Ausubel, F. M., Rosner, B. & Michel, J. L. Inactivation of the α C protein antigen gene, *bca*, by a novel shuttle/suicide vector results in attenuation of virulence and immunity in group B *Streptococcus*. *Proc. Natl. Acad. Sci. USA* **94**, 13251-13256, 1997.
