# Supplementary material for: Environmental pH and peptide signaling control virulence of Streptococcus pyogenes via a quorum-sensing pathway
Source: Nat Commun. 2019 Jun 13;10:2586. doi: 10.1038/s41467-019-10556-8 (PMC6565748; doi:10.1038/s41467-019-10556-8)
Supplement: Supplementary file 3 — Reporting Summary [file 41467_2019_10556_MOESM3_ESM.pdf]

nature research

Corresponding author(s): Muthiah Kumaraswami

Reporting Summary

Nature Research wishes to improve the reproducibility of the work that we publish. This form provides structure for consistency and transparency in reporting. For further information on Nature Research policies, see Authors & Referees and the Editorial Policy Checklist.

Statistical parameters

When statistical analyses are reported, confirm that the following items are present in the relevant location (e.g. figure legend, table legend, main text, or Methods section).

n/a	Confirmed	
☐	☒	The exact sample size (n) for each experimental group/condition, given as a discrete number and unit of measurement
☐	☒	An indication of whether measurements were taken from distinct samples or whether the same sample was measured repeatedly
☐	☒	The statistical test(s) used AND whether they are one- or two-sided<br>*Only common tests should be described solely by name; describe more complex techniques in the Methods section.*
☒	☐	A description of all covariates tested
☒	☐	A description of any assumptions or corrections, such as tests of normality and adjustment for multiple comparisons
☒	☐	A full description of the statistics including central tendency (e.g. means) or other basic estimates (e.g. regression coefficient) AND variation (e.g. standard deviation) or associated estimates of uncertainty (e.g. confidence intervals)
☒	☐	For null hypothesis testing, the test statistic (e.g. F, t, r) with confidence intervals, effect sizes, degrees of freedom and P value noted<br>*Give P values as exact values whenever suitable.*
☒	☐	For Bayesian analysis, information on the choice of priors and Markov chain Monte Carlo settings
☒	☐	For hierarchical and complex designs, identification of the appropriate level for tests and full reporting of outcomes
☒	☐	Estimates of effect sizes (e.g. Cohen's d, Pearson's r), indicating how they were calculated
☐	☒	Clearly defined error bars<br>*State explicitly what error bars represent (e.g. SD, SE, CI)*

Our web collection on statistics for biologists may be useful.

Software and code

Policy information about availability of computer code

Data collection	Not applicable.
Data analysis	Not applicable.

For manuscripts utilizing custom algorithms or software that are central to the research but not yet described in published literature, software must be made available to editors/reviewers upon request. We strongly encourage code deposition in a community repository (e.g. GitHub). See the Nature Research guidelines for submitting code & software for further information.

Data

Policy information about availability of data

All manuscripts must include a data availability statement. This statement should provide the following information, where applicable:

- Accession codes, unique identifiers, or web links for publicly available datasets
- A list of figures that have associated raw data
- A description of any restrictions on data availability

The structure of RopB-CTD-SIP complex was determined by molecular replacement. The final model was submitted to Protein Data Bank (PDB) data base and the accession number is provided in the manuscript.

Field-specific reporting

Please select the best fit for your research. If you are not sure, read the appropriate sections before making your selection.

☒ Life sciences ☐ Behavioural & social sciences ☐ Ecological, evolutionary & environmental sciences

For a reference copy of the document with all sections, see nature.com/authors/policies/ReportingSummary-flat.pdf

Life sciences study design

All studies must disclose on these points even when the disclosure is negative.

Sample size	The 1x10^7 CFU inoculum used for the mouse infection studies was determined by Probit analysis (XLSTAT2011) of survival data generated from a dose-escalation study with virulence reference strain MGAS10870. A power calculation for the log-rank test (www.statstodo.com) predicted that a sample size of 20 mice per strain treatment group was needed to generate statistically significant results when using the pilot study survival rates and parameters alpha=0.05, beta=0.8, and r=1. Power calculations for other virulence experiments described in this study were performed in a manner as described above.
Data exclusions	No data was excluded from the analyses.
Replication	Biochemical experiments, transcript level analyses, immunoblotting, and protease activity assays were replicated successfully 3 times. Confocal microscopy experiments were conducted on three biological replicates and representative image is shown in the figure.
Randomization	Random allocation of strains is not relevant to our study.
Blinding	Strains were assigned numbers in the mouse infection studies. The exact identity of these strains was unknown until the experimental part and the analyses were completed.

Reporting for specific materials, systems and methods

Materials & experimental systems

n/a	Involved in the study	
☒	☐	Unique biological materials
☐	☒	Antibodies
☒	☐	Eukaryotic cell lines
☒	☐	Palaeontology
☐	☒	Animals and other organisms
☒	☐	Human research participants

Methods

n/a	Involved in the study	
☒	☐	ChIP-seq
☒	☐	Flow cytometry
☒	☐	MRI-based neuroimaging

Antibodies

Antibodies used	A custom-made rabbit affinity-purified polyclonal antibody (Pacific Immunology) was used to detect the secreted SpeB protein. The anti-SpeB antibody was made against a purified recombinant mature SpeB protease. A 1:50000 dilution of this antibody was used. A generic sheep anti-rabbit IgG conjugated with horse radish peroxidase was used as a secondary antibody for chemiluminescence-based detection. The secondary antibody was used at 1:50000 dilution.
Validation	The custom-made anti-SpeB antibody did not cross-react against any other protein made by emm3 Group A streptococci.

Animals and other organisms

Policy information about studies involving animals; ARRIVE guidelines recommended for reporting animal research

Laboratory animals	- species: Mus musculus<br>- strain: CD1 (Envigo Laboratories)<br>- sex: female<br>- age: 4 weeks
Wild animals	The study did involve the use of wild animals

Field-collected samples

The study did not involve samples collected from the field
